# Exploring plasma microRNA profiling and signaling pathways in comorbidity-free sickle cell anemia: a pilot case-control study

**DOI:** 10.1186/s13104-026-07765-y

**Published:** 2026-03-18

**Authors:** Kabir Olaniran, Ronak Lakhia, Scott Krinsky, Sagar Nigwekar, Susan Hedayati

**Affiliations:** 1 Division Of Nephrology, Department of Internal Medicine, University of Texas Southwestern Medical Center, Dallas, TX USA; 2 Nephrology Division, Department of Medicine, Massachusetts General Hospital, Harvard Medical School, Boston, MA USA; 3 Division of Nephrology and Hypertension, Department of Medicine, Stony Brook University School of Medicine, Stony Brook, NY USA

**Keywords:** Sickle cell anemia, MicroRNA, Pilot Study

## Abstract

**Objective:**

Sickle cell anemia (SCA) is associated with substantial end‑organ morbidity, yet the circulating microRNA (miRNA) landscape in clinically stable, comorbidity‑free adults with SCA is poorly defined. This pilot case-control study with a limited sample size aimed to characterize a baseline plasma miRNA signature of SCA by comparing comorbidity‑free adults with SCA and strictly matched controls, with findings intended to be hypothesis‑generating.

**Results:**

Using the Mass General Brigham Biobank, we identified 4 adults with HbSS SCA and 12 HbAA controls, matched 1:3 by age, sex, and estimated glomerular filtration rate. All participants were free of diabetes, hypertension, cardiovascular disease, chronic kidney disease, and proteinuria. Plasma small RNA sequencing identified 41 differentially expressed miRNAs in SCA versus controls (2 upregulated, 39 downregulated; *p* < 0.05). These miRNAs mapped to 99 enriched KEGG pathways, including PI3K/Akt, MAPK, and Hippo signaling, which are implicated in endothelial dysfunction, vasculopathy, and organ complications relevant to SCA. These preliminary, hypothesis‑generating findings from a small pilot cohort support the presence of subclinical vascular and inflammatory pathway dysregulation in comorbidity‑free SCA and provide candidate miRNAs and pathways for future validation in larger, independent cohorts. These results represent unvalidated discovery signals and should not be interpreted as a definitive miRNA profile.

**Supplementary Information:**

The online version contains supplementary material available at 10.1186/s13104-026-07765-y.

## Introduction

Sickle cell anemia (SCA) is the most common monogenic disorder in the world and remains a major public health challenge [1]. Despite therapeutic advances, SCA is still associated with substantially reduced life expectancy, largely due to cumulative end-organ damage such as cerebral infarcts, pulmonary hypertension, heart failure, and chronic kidney disease [2].

Genetic and molecular modifiers of these complications are an active area of investigation [3]. MicroRNAs (miRNAs) are small non‑coding RNAs that regulate gene expression post‑transcriptionally and have emerged as important candidates in vascular, inflammatory, and fibrotic pathways [4]. Prior SCA miRNA studies have focused mainly on intracellular or red‑blood‑cell miRNAs, or on circulating miRNAs in patients with severe or established complications [5, 6]. In contrast, the circulating plasma miRNA landscape in comorbidity‑free adults with SCA is poorly characterized.

Understanding baseline plasma miRNA profiles in co-morbidity free SCA may help reveal subclinical pathway dysregulation that precedes overt organ damage. It is important to note that SCA is characterized by chronic hemolysis, endothelial activation, and subclinical organ stress even in the absence of diagnosed comorbidities; therefore, “comorbidity‑free” in this context refers specifically to the absence of documented chronic end‑organ complications rather than disease inactivity [1, 3]. This pilot case-control study compared circulating plasma miRNA expression in comorbidity‑free adults with SCA and strictly matched HbAA controls, with the hypothesis that SCA would be associated with a distinct plasma miRNA signature despite the absence of overt clinical disease.

## Methods

### Study design and data source

This cross‑sectional pilot study used data and banked plasma samples from the Mass General Brigham (MGB) Biobank (2005–2018), a repository of biospecimens collected with informed consent and linked to electronic health records from MGB‑affiliated hospitals. The MGB Biobank has been described previously [7]. This study was approved by the MGB and University of Texas Southwestern (UTSW) Institutional Review Boards (UTSW IRB #STU-2020-1053).

### Participant selection and matching

At the time of sample collection, inclusion criteria were: (1) self‑identified Black race, (2) age ≥ 18 years, (3) estimated glomerular filtration rate (eGFR) > 60 mL/min/1.73 m², and (4) available urine protein studies. Exclusion criteria included age ≥ 65 years or any documented history of diabetes mellitus, hypertension, cardiovascular disease, heart failure, pulmonary hypertension, chronic kidney disease, malignancy, transplantation, autoimmune disease, or proteinuria. Hemoglobin phenotype (HbSS [SCA/cases] vs. HbAA [normal hemoglobin phenotype/controls]) was confirmed by chart review of pathologist‑interpreted hemoglobin electrophoresis. Cases and controls were matched 1:3 on age (± 5 years), sex, and eGFR (± 20 mL/min/1.73 m²).

### Plasma miRNA sequencing and processing

Frozen plasma aliquots (0.5 mL per participant) were shipped to a commercial laboratory (LC Sciences, Houston, USA) for small RNA sequencing [8]. Total RNA was extracted using Trizol reagent, and RNA integrity was assessed by Bioanalyzer 2100 (Agilent, CA, USA); samples with RNA integrity number (RIN) > 7.0 were retained. One microgram of total RNA per sample was used to construct small RNA libraries with the TruSeq Small RNA Sample Preparation Kit (Illumina, San Diego, USA), followed by 50‑bp single‑end sequencing on an Illumina HiSeq 2500 platform.

Raw reads were processed with the ACGT101‑miR pipeline to remove adapter dimers, low‑quality and low‑complexity reads, and common RNA families (rRNA, tRNA, snRNA, snoRNA and repeats). Unique sequences 18–26 nucleotides in length were mapped to human miRBase version 22.0 using BLAST to identify known and putative novel 3p‑ and 5p‑derived miRNAs. miRNA counts were normalized by dividing each miRNA count by a sample‑specific library size parameter.

### Target prediction and pathway analysis

Two target‑prediction algorithms (TargetScan and miRanda 3.3a) were used to identify putative miRNA binding sites; only overlapping predicted targets were retained. Kyoto Encyclopedia of Genes and Genomes (KEGG) pathway enrichment analysis was then performed on the target gene set using a hypergeometric test, and pathways with *p* < 0.05 were considered significantly enriched.

### Statistical analyses

Differential expression of plasma miRNAs between SCA (HbSS) cases and HbAA controls was assessed using the non‑parametric Wilcoxon rank‑sum test on normalized counts, with *p* < 0.05 defining statistical significance for this exploratory pilot analysis. The non-parametric Wilcoxon rank-sum test was selected because it does not assume normality of the underlying distribution, making it appropriate for small-sample comparisons where parametric assumptions cannot be reliably verified; however, statistical power remains limited at this sample size, and results should be interpreted as exploratory.

## Results

Of 196 biobank participants with hemoglobin electrophoresis and available plasma (182 HbAA, 14 SCA), 80 HbAA and 4 SCA individuals met all inclusion and exclusion criteria. After 1:3 matching on age, sex, and eGFR, 4 SCA cases and 12 HbAA controls were included in the final analytic cohort.

Compared with HbAA controls, SCA participants were slightly younger (32 ± 7 vs. 36 ± 8 years) and had higher mean eGFR (144 ± 13 vs. 118 ± 18 mL/min/1.73 m²). Females comprised 25% of both groups. Among SCA participants, median hemoglobin was 8.3 g/dL (interquartile range [IQR] 8.1 to 8.9), and median total bilirubin was 1.8 mg/dL (IQR 1.6 to 4.5). One SCA participant was receiving hydroxyurea at the time of sample collection.

**Fig. 1:**
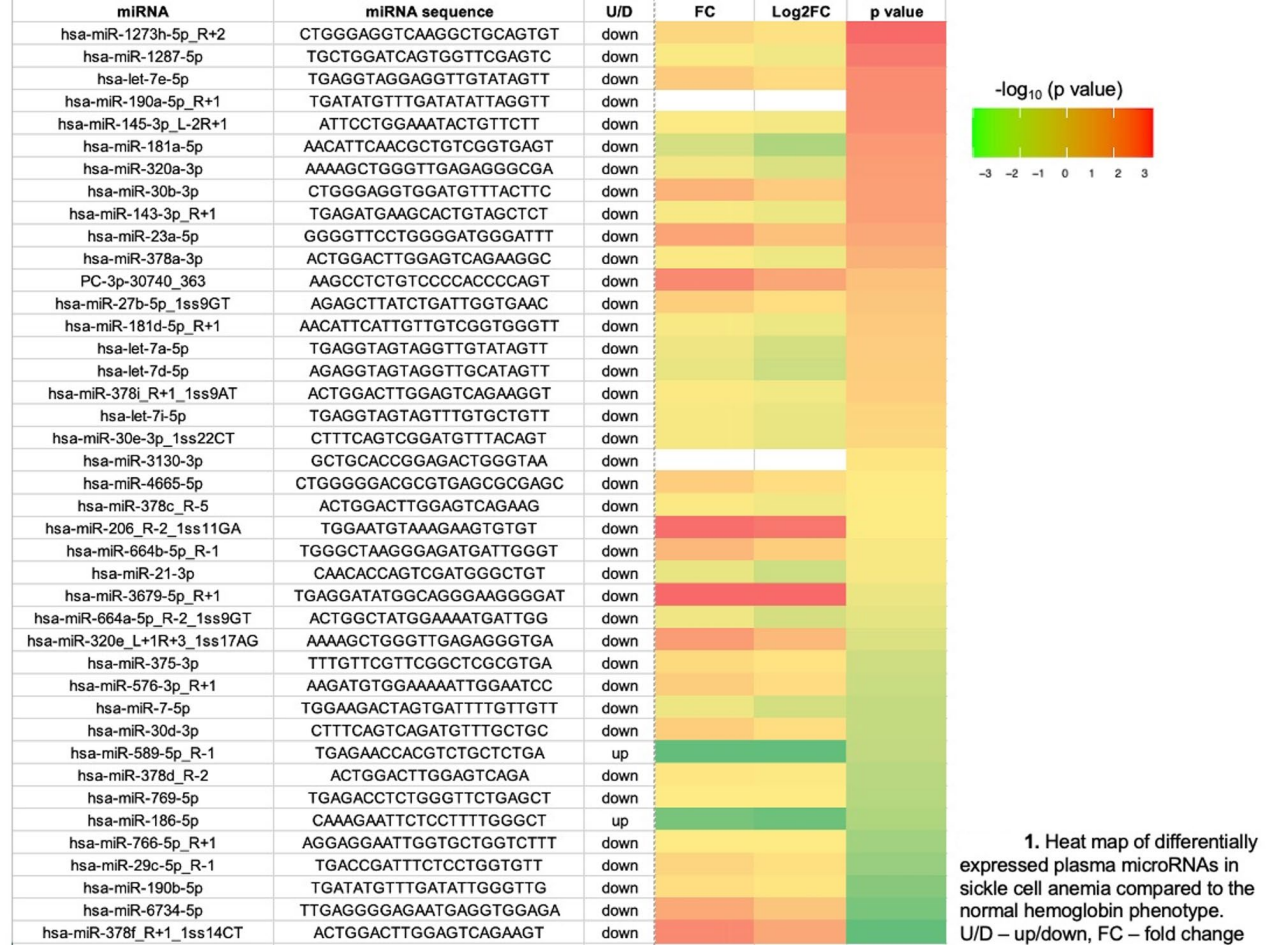
Heat map of differentially expressed plasma microRNAs in sickle cell anemia compared to the normal hemoglobin phenotype

**Fig. 2:**
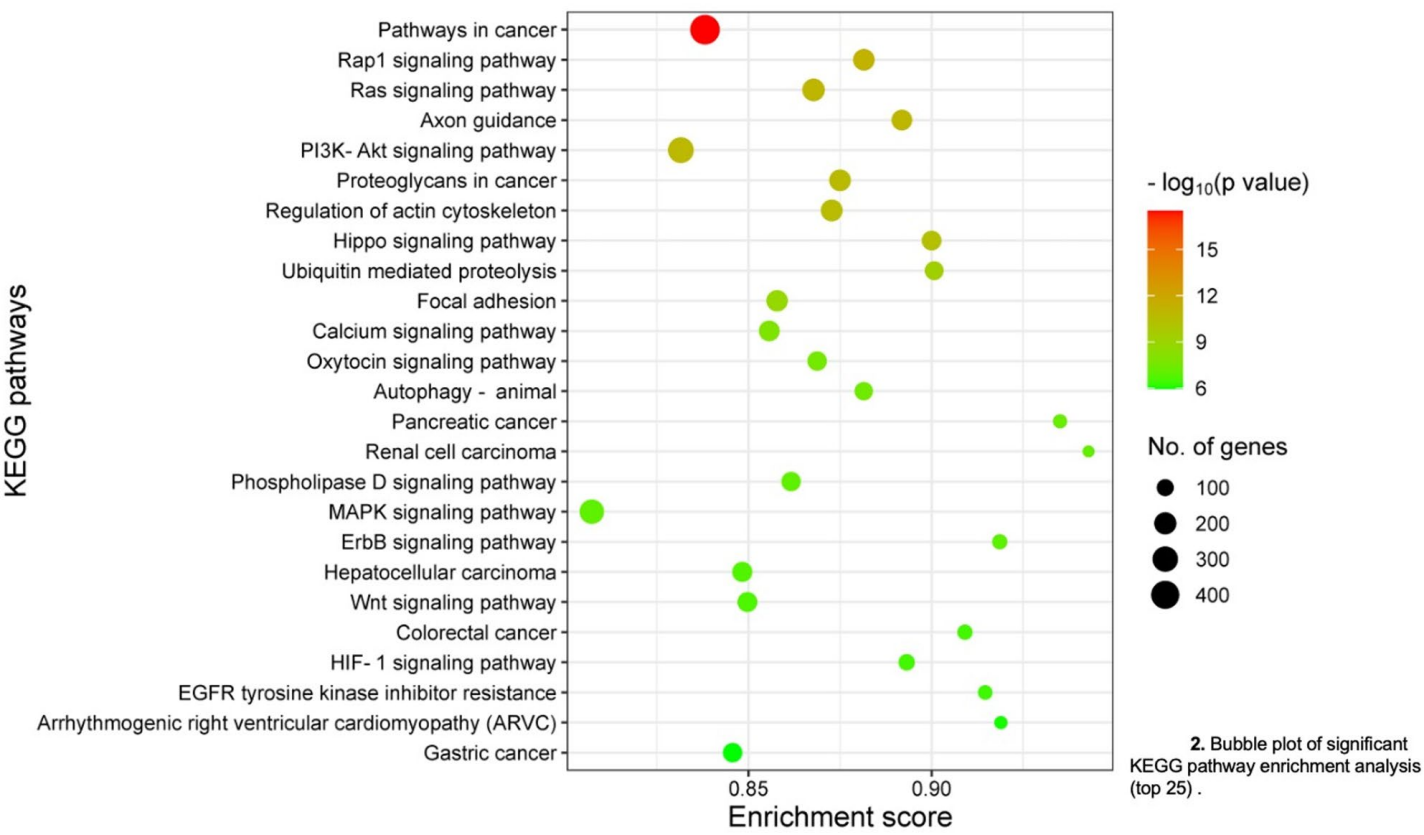
Bubble plot of significant KEGG pathway enrichment analysis (top 25)

Plasma small RNA sequencing identified 41 differentially expressed miRNAs between SCA and HbAA (Wilcoxon *p* < 0.05), including 2 with increased and 39 with reduced abundance in SCA (Fig. 1; Supplemental Table 1). Target‑gene prediction followed by KEGG enrichment analysis mapped these miRNAs to 99 significantly enriched signaling and metabolic pathways (*p* < 0.05), including PI3K/Akt, MAPK, TGF‑β, and Hippo pathways (Fig. 2; Supplemental Table 2). Many of these pathways are implicated in endothelial activation, vasculopathy, and cardiorenal and pulmonary complications relevant to SCA.

## Discussion

In this cross‑sectional pilot study of comorbidity‑free adults, sickle cell anemia (SCA) was associated with a preliminary pattern of differentially expressed plasma microRNAs compared with strictly matched HbAA controls. Given the limited sample size (*n* = 4 SCA cases), these findings should be interpreted as hypothesis‑generating and represent preliminary signals rather than a definitive miRNA profile. Although participants had no diagnosed chronic comorbidities, several of these microRNAs have been linked in prior work to endothelial inflammation, oxidative stress, and cerebrovascular ischemia (e.g. hsa‑let‑7e‑5p, hsa‑miR‑181a‑5p, hsa‑miR‑7‑5p) [9–11], as well as cardiomyopathy, pulmonary hypertension, and chronic kidney disease (e.g. hsa‑miR‑320a‑3p, hsa‑miR‑30b‑3p, hsa‑miR‑21‑3p, hsa‑miR‑1287‑5p, hsa‑miR‑378a‑3p) [12–17]. These observations raise the possibility that, even in clinically comorbidity-free SCA, circulating miRNAs may reflect subclinical vascular and inflammatory activation relevant to future organ damage.

Notably, SCA participants had a higher mean eGFR compared with controls (144 vs. 118 mL/min/1.73 m²). Although all participants were free of chronic kidney disease and proteinuria, this elevated eGFR may reflect glomerular hyperfiltration, a recognized early manifestation of sickle cell nephropathy driven by chronic hemolysis, medullary ischemia, and prostaglandin‑mediated increases in renal plasma flow, which can occur in the absence of overt renal disease [18, 19]. This observation is consistent with the concept that subclinical organ stress is present even in comorbidity‑free SCA and underscores the potential relevance of the differentially expressed miRNAs identified in this study to early renal pathophysiology.

Pathway analyses provided convergent support for this interpretation. The differentially expressed miRNAs mapped to multiple enriched KEGG pathways, including PI3K/Akt, MAPK, TGF‑β, Hippo, and other signaling networks previously implicated in ischemic stroke, heart failure, pulmonary hypertension, and chronic kidney disease [20–27]. While these findings do not establish causality, they highlight specific miRNAs and pathways that merit targeted evaluation in future, adequately powered validation studies.

### Limitations

This study has important limitations. The sample size was very small (4 SCA vs. 12 controls), increasing the risk of false‑positive findings and effect‑size inflation, and precluding robust adjustment for confounding; results should therefore be viewed as preliminary signals from an exploratory pilot analysis. Alpha‑thalassemia and other genetic modifiers were not assessed, and there was no independent validation cohort or longitudinal follow‑up to link miRNA profiles with incident complications. One SCA participant was receiving hydroxyurea at the time of sample collection. Hydroxyurea is known to modulate miRNA expression profiles, including upregulation of fetal hemoglobin‑associated miRNAs and alteration of inflammatory signaling pathways [28], and its use in one of four cases represents a significant potential confounder that limits the interpretability of individual miRNA‑level findings. Additionally, data on vaso‑occlusive crisis frequency, transfusion history, fetal hemoglobin levels, and inflammatory markers (e.g., lacate dehydrogenase, ferritin, reticulocyte count) were not systematically available in the biobank repository at the time of sample collection, and the absence of these data precludes a comprehensive characterization of disease activity in the SCA cohort. Future validation studies should incorporate these clinical parameters. Despite these constraints, the strict exclusion of comorbidities and 1:3 matching on age, sex, and kidney function provide a clean, hypothesis‑generating comparison of baseline plasma miRNA profiles in SCA versus HbAA. However, these findings represent unvalidated discovery signals from small RNA sequencing and have not been confirmed by orthogonal methods such as RT-qPCR. The absence of technical validation limits the robustness of individual miRNA‑level findings, and these results should not be interpreted as candidate biomarkers ready for clinical development. Rather, they provide a preliminary basis for hypothesis generation and prioritization of targets for future investigation.

## Conclusions

In conclusion, comorbidity‑free adults with SCA demonstrated differential expression of circulating plasma miRNAs that map to pathways involved in major SCA complications. These preliminary, unvalidated data should be interpreted as hypothesis‑generating but may help prioritize candidate miRNAs and signaling pathways for independent validation in larger, adequately powered longitudinal SCA cohorts prior to any consideration for biomarker development. Technical validation by orthogonal methods such as RT‑qPCR will be an essential next step.

## Supplementary Information

Below is the link to the electronic supplementary material.

Supplementary Material 1.

## Data Availability

The microRNA data from sickle cell anemia and normal hemoglobin phenotype patients have been submitted to the Gene Expression Omnibus under accession number GSE285580.Additional information will be provided upon reasonable request by emails to the corresponding author.

## Abbreviations

PI3K/Akt: Phosphatidylinositol 3-kinase (PI3K) and Protein Kinase B (Akt)
MAPK: Mitogen-Activated Protein Kinase
TGF‑β: Transforming Growth Factor-beta
