# Supplementary material for: Exploring plasma microRNA profiling and signaling pathways in comorbidity-free sickle cell anemia: a pilot case-control study
Source: BMC Res Notes. 2026 Mar 18;19:189. doi: 10.1186/s13104-026-07765-y (PMC13122893; doi:10.1186/s13104-026-07765-y)
Supplement: Supplementary file 1 — Supplementary Material 1. [file 13104_2026_7765_MOESM1_ESM.docx]

**Supplemental table 1.** Differentially expressed plasma microRNAs in sickle cell anemia compared to normal hemoglobin phenotype

| **miRNA** | **miRNA sequence** | **U/D** | **FC** | **Log2FC** | **p value** |
| --- | --- | --- | --- | --- | --- |
| hsa-miR-1273h-5p_R + 2 | CTGGGAGGTCAAGGCTGCAGTGT | down | 0.21 | -2.25 | 5.80E-03 |
| hsa-miR-1287-5p | TGCTGGATCAGTGGTTCGAGTC | down | 0.29 | -1.77 | 8.48E-03 |
| hsa-let-7e-5p | TGAGGTAGGAGGTTGTATAGTT | down | 0.19 | -2.39 | 1.14E-02 |
| hsa-miR-190a-5p_R + 1 | TGATATGTTTGATATATTAGGTT | down | -inf | -inf | 1.17E-02 |
| hsa-miR-145-3p_L-2R + 1 | ATTCCTGGAAATACTGTTCTT | down | 0.28 | -1.81 | 1.18E-02 |
| hsa-miR-181a-5p | AACATTCAACGCTGTCGGTGAGT | down | 0.69 | -0.53 | 1.35E-02 |
| hsa-miR-320a-3p | AAAAGCTGGGTTGAGAGGGCGA | down | 0.39 | -1.35 | 1.42E-02 |
| hsa-miR-30b-3p | CTGGGAGGTGGATGTTTACTTC | down | 0.15 | -2.69 | 1.45E-02 |
| hsa-miR-143-3p_R + 1 | TGAGATGAAGCACTGTAGCTCT | down | 0.31 | -1.68 | 1.46E-02 |
| hsa-miR-23a-5p | GGGGTTCCTGGGGATGGGATTT | down | 0.13 | -2.95 | 1.61E-02 |
| hsa-miR-378a-3p | ACTGGACTTGGAGTCAGAAGGC | down | 0.30 | -1.72 | 1.76E-02 |
| PC-3p-30740_363 | AAGCCTCTGTCCCCACCCCAGT | down | 0.09 | -3.51 | 2.02E-02 |
| hsa-miR-27b-5p_1ss9GT | AGAGCTTATCTGATTGGTGAAC | down | 0.20 | -2.33 | 2.07E-02 |
| hsa-miR-181d-5p_R + 1 | AACATTCATTGTTGTCGGTGGGTT | down | 0.31 | -1.67 | 2.15E-02 |
| hsa-let-7a-5p | TGAGGTAGTAGGTTGTATAGTT | down | 0.44 | -1.20 | 2.17E-02 |
| hsa-let-7d-5p | AGAGGTAGTAGGTTGCATAGTT | down | 0.47 | -1.09 | 2.20E-02 |
| hsa-miR-378i_R + 1_1ss9AT | ACTGGACTTGGAGTCAGAAGGT | down | 0.29 | -1.79 | 2.21E-02 |
| hsa-let-7i-5p | TGAGGTAGTAGTTTGTGCTGTT | down | 0.32 | -1.63 | 2.32E-02 |
| hsa-miR-30e-3p_1ss22CT | CTTTCAGTCGGATGTTTACAGT | down | 0.33 | -1.61 | 2.34E-02 |
| hsa-miR-3130-3p | GCTGCACCGGAGACTGGGTAA | down | -inf | -inf | 2.58E-02 |
| hsa-miR-4665-5p | CTGGGGGACGCGTGAGCGCGAGC | down | 0.20 | -2.35 | 2.67E-02 |
| hsa-miR-378c_R-5 | ACTGGACTTGGAGTCAGAAG | down | 0.29 | -1.78 | 2.71E-02 |
| hsa-miR-206_R-2_1ss11GA | TGGAATGTAAAGAAGTGTGT | down | 0.04 | -4.51 | 2.71E-02 |
| hsa-miR-664b-5p_R-1 | TGGGCTAAGGGAGATGATTGGGT | down | 0.16 | -2.65 | 2.77E-02 |
| hsa-miR-21-3p | CAACACCAGTCGATGGGCTGT | down | 0.47 | -1.08 | 2.79E-02 |
| hsa-miR-3679-5p_R + 1 | TGAGGATATGGCAGGGAAGGGGAT | down | 0.04 | -4.83 | 2.95E-02 |
| hsa-miR-664a-5p_R-2_1ss9GT | ACTGGCTATGGAAAATGATTGG | down | 0.42 | -1.24 | 3.04E-02 |
| hsa-miR-320e_L + 1R+3_1ss17AG | AAAAGCTGGGTTGAGAGGGTGA | down | 0.12 | -3.09 | 3.17E-02 |
| hsa-miR-375-3p | TTTGTTCGTTCGGCTCGCGTGA | down | 0.22 | -2.21 | 3.34E-02 |
| hsa-miR-576-3p_R + 1 | AAGATGTGGAAAAATTGGAATCC | down | 0.19 | -2.36 | 3.42E-02 |
| hsa-miR-7-5p | TGGAAGACTAGTGATTTTGTTGTT | down | 0.44 | -1.19 | 3.50E-02 |
| hsa-miR-30d-3p | CTTTCAGTCAGATGTTTGCTGC | down | 0.20 | -2.34 | 3.51E-02 |
| hsa-miR-589-5p_R-1 | TGAGAACCACGTCTGCTCTGA | up | 1.93 | 0.95 | 3.54E-02 |
| hsa-miR-378d_R-2 | ACTGGACTTGGAGTCAGA | down | 0.23 | -2.09 | 3.64E-02 |
| hsa-miR-769-5p | TGAGACCTCTGGGTTCTGAGCT | down | 0.24 | -2.05 | 3.69E-02 |
| hsa-miR-186-5p | CAAAGAATTCTCCTTTTGGGCT | up | 1.71 | 0.77 | 3.77E-02 |
| hsa-miR-766-5p_R + 1 | AGGAGGAATTGGTGCTGGTCTTT | down | 0.24 | -2.04 | 3.95E-02 |
| hsa-miR-29c-5p_R-1 | TGACCGATTTCTCCTGGTGTT | down | 0.21 | -2.28 | 4.08E-02 |
| hsa-miR-190b-5p | TGATATGTTTGATATTGGGTTG | down | 0.22 | -2.18 | 4.35E-02 |
| hsa-miR-6734-5p | TTGAGGGGAGAATGAGGTGGAGA | down | 0.14 | -2.86 | 4.52E-02 |
| hsa-miR-378f_R + 1_1ss14CT | ACTGGACTTGGAGTCAGAAGT | down | 0.09 | -3.47 | 4.82E-02 |

**Supplemental table 2.** Significant KEGG metabolic signaling pathways in sickle cell anemia

| **Pathway** | **Enrichment** | **P value** | **Gene number** |
| --- | --- | --- | --- |
| Pathways in cancer | 0.8381295 | 3.08E-18 | 466 |
| Rap1 signaling pathway | 0.88151659 | 5.88E-12 | 186 |
| Ras signaling pathway | 0.8677686 | 8.75E-12 | 210 |
| Axon guidance | 0.89189189 | 9.77E-12 | 165 |
| PI3K-Akt signaling pathway | 0.8315508 | 1.27E-11 | 311 |
| Proteoglycans in cancer | 0.875 | 1.87E-11 | 189 |
| Regulation of actin cytoskeleton | 0.87272727 | 2.21E-11 | 192 |
| Hippo signaling pathway | 0.9 | 4.11E-11 | 144 |
| Ubiquitin mediated proteolysis | 0.90070922 | 5.28E-10 | 127 |
| Focal adhesion | 0.85781991 | 1.99E-09 | 181 |
| Calcium signaling pathway | 0.8556701 | 1.40E-08 | 166 |
| Oxytocin signaling pathway | 0.86875 | 2.66E-08 | 139 |
| Autophagy - animal | 0.88148148 | 4.19E-08 | 119 |
| Pancreatic cancer | 0.93506494 | 6.64E-08 | 72 |
| Renal cell carcinoma | 0.94285714 | 9.60E-08 | 66 |
| Phospholipase D signaling pathway | 0.86163522 | 1.04E-07 | 137 |
| MAPK signaling pathway | 0.80722892 | 1.10E-07 | 268 |
| ErbB signaling pathway | 0.91860465 | 1.29E-07 | 79 |
| Hepatocellular carcinoma | 0.84831461 | 2.11E-07 | 151 |
| Wnt signaling pathway | 0.84971098 | 2.47E-07 | 147 |
| Colorectal cancer | 0.90909091 | 3.47E-07 | 80 |
| HIF-1 signaling pathway | 0.89320388 | 3.60E-07 | 92 |
| EGFR tyrosine kinase inhibitor resistance | 0.91463415 | 4.36E-07 | 75 |
| Arrhythmogenic right ventricular cardiomyopathy (ARVC) | 0.91891892 | 9.84E-07 | 68 |
| Gastric cancer | 0.84567901 | 1.13E-06 | 137 |
| Signaling pathways regulating pluripotency of stem cells | 0.84868421 | 1.55E-06 | 129 |
| Apelin signaling pathway | 0.85106383 | 2.67E-06 | 120 |
| Long-term depression | 0.92307692 | 2.98E-06 | 60 |
| Fc gamma R-mediated phagocytosis | 0.88421053 | 3.22E-06 | 84 |
| FoxO signaling pathway | 0.85 | 3.36E-06 | 119 |
| Inflammatory mediator regulation of TRP channels | 0.87378641 | 4.63E-06 | 90 |
| Sphingolipid signaling pathway | 0.84962406 | 6.23E-06 | 113 |
| Prostate cancer | 0.87128713 | 7.74E-06 | 88 |
| Thyroid hormone signaling pathway | 0.84848485 | 7.84E-06 | 112 |
| Phosphatidylinositol signaling system | 0.86538462 | 1.14E-05 | 90 |
| Melanogenesis | 0.85981308 | 1.64E-05 | 92 |
| cAMP signaling pathway | 0.80630631 | 1.66E-05 | 179 |
| cGMP-PKG signaling pathway | 0.82183908 | 1.77E-05 | 143 |
| Chronic myeloid leukemia | 0.88607595 | 1.84E-05 | 70 |
| Prolactin signaling pathway | 0.89189189 | 1.94E-05 | 66 |
| Glutamatergic synapse | 0.84745763 | 2.68E-05 | 100 |
| Choline metabolism in cancer | 0.85321101 | 2.90E-05 | 93 |
| Transcriptional misregulation in cancer | 0.81025641 | 3.11E-05 | 158 |
| Breast cancer | 0.82208589 | 3.15E-05 | 134 |
| Vascular smooth muscle contraction | 0.83211679 | 4.23E-05 | 114 |
| VEGF signaling pathway | 0.90163934 | 4.72E-05 | 55 |
| Central carbon metabolism in cancer | 0.88405797 | 7.63E-05 | 61 |
| AMPK signaling pathway | 0.83076923 | 7.72E-05 | 108 |
| Gap junction | 0.84848485 | 0 | 84 |
| Neurotrophin signaling pathway | 0.83064516 | 0 | 103 |
| Jak-STAT signaling pathway | 0.80952381 | 0 | 136 |
| Chemokine signaling pathway | 0.79439252 | 0 | 170 |
| AGE-RAGE signaling pathway in diabetic complications | 0.83478261 | 0 | 96 |
| Melanoma | 0.86842105 | 0 | 66 |
| Insulin signaling pathway | 0.81290323 | 0 | 126 |
| GnRH signaling pathway | 0.84536082 | 0 | 82 |
| Circadian entrainment | 0.84158416 | 0 | 85 |
| Endometrial cancer | 0.8852459 | 0 | 54 |
| Glioma | 0.86075949 | 0 | 68 |
| p53 signaling pathway | 0.86486486 | 0 | 64 |
| Bacterial invasion of epithelial cells | 0.85897436 | 0 | 67 |
| Dopaminergic synapse | 0.8125 | 0 | 117 |
| Relaxin signaling pathway | 0.81428571 | 0 | 114 |
| Platelet activation | 0.81617647 | 0 | 111 |
| SNARE interactions in vesicular transport | 0.94117647 | 0 | 32 |
| Hepatitis B | 0.79775281 | 0 | 142 |
| Acute myeloid leukemia | 0.86764706 | 0 | 59 |
| Hedgehog signaling pathway | 0.89795918 | 0 | 44 |
| Morphine addiction | 0.83333333 | 0 | 85 |
| Parathyroid hormone synthesis, secretion and action | 0.8220339 | 0 | 97 |
| Platinum drug resistance | 0.85526316 | 0 | 65 |
| Apoptosis | 0.80392157 | 0 | 123 |
| Adrenergic signaling in cardiomyocytes | 0.80128205 | 0 | 125 |
| Lysosome | 0.80714286 | 0 | 113 |
| Inositol phosphate metabolism | 0.84810127 | 0 | 67 |
| Aldosterone-regulated sodium reabsorption | 0.91891892 | 0 | 34 |
| Non-small cell lung cancer | 0.85135135 | 0 | 63 |
| Lysine degradation | 0.86153846 | 0 | 56 |
| Circadian rhythm | 0.93548387 | 0 | 29 |
| Leukocyte transendothelial migration | 0.81355932 | 0 | 96 |
| Glycine, serine and threonine metabolism | 0.90243902 | 0 | 37 |
| Amino sugar and nucleotide sugar metabolism | 0.88 | 0 | 44 |
| Cysteine and methionine metabolism | 0.88 | 0 | 44 |
| Small cell lung cancer | 0.82178218 | 0 | 83 |
| Hippo signaling pathway - multiple species | 0.93333333 | 0 | 28 |
| Adherens junction | 0.8375 | 0 | 67 |
| Insulin resistance | 0.80645161 | 0 | 100 |
| Cushing syndrome | 0.78531073 | 0 | 139 |
| Fluid shear stress and atherosclerosis | 0.79333333 | 0 | 119 |
| Gastric acid secretion | 0.83544304 | 0 | 66 |
| Cholinergic synapse | 0.80327869 | 0 | 98 |
| Purine metabolism | 0.79562044 | 0 | 109 |
| TGF-beta signaling pathway | 0.81372549 | 0 | 83 |
| Bladder cancer | 0.88372093 | 0 | 38 |
| Cell cycle | 0.79844961 | 0 | 103 |
| Biosynthesis of unsaturated fatty acids | 0.92857143 | 0 | 26 |
| Apoptosis - multiple species | 0.90909091 | 0 | 30 |
| T cell receptor signaling pathway | 0.80530973 | 0 | 91 |
| Fanconi anemia pathway | 0.85714286 | 0 | 48 |
